# A Case of Successful Operation for the Exsection of Two Carious Ribs, and the Lower Portion of the Sternum

**Published:** 1850-02

**Authors:** George McClellan

**Affiliations:** Philadelphia


### MEDICAL EXAMINER,

AND

### RECORD OF MEDICAL SCIENCE.

NEW SERIES.—NO. LXII.—FEBRUARY, 1850.

### ORIGINAL COMMUNICATIONS.

*A case of Successful Operation for the Exsection of two Carious Ribs, and the lower portion of the Sternum.* By the late GEORGE McCLELLAN, M. D., of Philadelphia.

To F. G. Smith, M. D.

*My Dear Dr.*—The following case was lately found among my father's papers, in the form of a letter to a medical friend. As it is an exceedingly important and interesting one, and as I do not think it has ever been published, I enclose it to you for insertion in the "Examiner." The operation was performed in the year 1829, upon Mr. John Uhler, of Lebanon, Pa., for many years a member of the State Legislature, and was merely referred to by me in a note to my father's work on Surgery, page 354, as I had not then found these particulars.

Within a few days I have seen Dr. J. W. Gloninger, of Lebanon, Mr. Uhler's family physician, who attended him after the operation, from whom I learned that the patient recovered in about six weeks, without an unfavorable symptom, and lived until the fall of 1849, when he died of apoplexy, at the age of 72 years. The bones, &c., removed by the operation, are now in my father's surgical collection.

Truly yours,

J. H. B. McClellan, M. D.

*Philadelphia,* January 19, 1850.

Philadelphia, January 27th, 1831.

To ——— ———, M. D.

*My Dear Sir,*—Herewith I enclose the autograph letter of a patient, in the history of whose case you appear to have taken a deep interest. The bony specimens, which were presented by his family after the operation, have just come to hand, and I will send them to you before you leave the city.

As near as I can judge, from a recollection of his appearance at the time of the operation, I should suppose that the patient was about fifty years old. He was a very large person and corpulent; and his chest was at least as full and broad as I recollect ever to have observed in the case of any other individual. This will account for the unusual length of the cartilages of the ribs which were excised, and for the extent of the incisions which I was obliged to make in the superincumbent soft parts.

In addition to the symptoms enumerated in the enclosed letter, I must not omit to state, that I was particularly struck by the appearance of great oppression and difficulty of respiration under which the patient had been laboring for a considerable period of time before I visited him. On passing a probe through a fistulous opening, situated a short distance from the right edge of the lower portion of the sternum, I was also surprised to find the two adjacent costal cartilages presenting hard and rough bony surfaces, along both of which I could convey the probe its whole length towards the right side. This circumstance immediately convinced me that the disease had originated from an ossification of those cartilages, and a consequent inflammation in or around them, which had finally produced a detachment of the perichondria, and a caries, if not a necrosis, of both bony substances.

I commenced the operation by carrying a deep incision from the edge of the sternum outwards, to the distance of seven or eight inches, along the intercostal space between the *fifth* and *sixth* ribs. The edges of this incision were then dissected up until the surfaces of both those ribs were fully exposed. They were evidently ossified cartilages, presenting the appearance of worm-eaten caries throughout their whole length. The perichondrium was completely detached from the external surface of both, and thickened by induration of the surrounding cellular substance. I encountered but little difficulty, therefore, in detaching this envelope and pushing it backwards from the upper and lower edges of each rib by the use of the handle, and occasionally the blade of a common scalpel. The attenuated intercostal muscles and tendinous fibres were of course detached along with those membranes. The bones were then left sufficiently bare to allow of the application of a Hay's saw, which I repeated twice on each rib. With a common elevator I then pried up the bones from their outer towards their sternal attachments, in accomplishing which, only two or three applications of the knife were required to cut away adhesions to the indurated mass of membranes and cellular tissue within. As the projecting extremities of the ribs did not appear perfectly sound, I extended the incision a little further outwards, and cut away about one and a half inches more from their substance. The lower portion of the sternum presenting a similar appearance of disease, I also enlarged the incision inwards, and sawed away a considerable portion of that bone. On examining the exposed surface, there appeared a convoluted bony plate, surrounded by some indurated cellular tissue and medullary looking matter, pressing upon the pleura costalis, and intruding upon the cavity of the chest. I mistook this at first for the extremity of the seventh rib, which I supposed had been turned inwards and upwards behind the corresponding cartilages of the fifth and sixth ribs. On a closer inspection, however, I became convinced that it proceeded from ossification of the sixth rib, which had attempted to set up a sequestrating process around the cartilage.

I succeeded very easily in prying up the whole of this mass with an elevator, which detached it clearly from the pleura, with the same kind of sound that is emitted from a birch log when it is stripped of its bark. The intercostal fibres, both muscular and tendinous, were chiefly removed along with the bony plate, and the pleura was then left thin as natural, rising and falling alternately, with the efforts at inspiration and expiration.

As hemorrhage from the torn branches of the intercostal and mammary arteries ceased almost instantaneously, I immediately closed up the incision in the integuments, and retained them in apposition by interrupted sutures and adhesive plasters. A broad compress and bandage completed the dressing, and I left the patient directly afterwards in the care of my friend, Dr. J. W. Gloninger.

In haste, I am truly your friend,

GEO. McCLELLAN.

